# Clinical value of ^68^Ga-pentixafor PET/CT in patients with primary aldosteronism and bilateral lesions: preliminary results of a single-centre study

**DOI:** 10.1186/s13550-024-01125-2

**Published:** 2024-07-04

**Authors:** Rui Zuo, Shuang Liu, Wenbo Li, Zhu Xia, Lu Xu, Hua Pang

**Affiliations:** Department of Nuclear Medicine, The First Affiliated Hospital of Chongqing Medical University, No. 1 Youyi Road, Chongqing, Chongqing, 400016 China

**Keywords:** Primary aldosteronism, ^68^Ga-Pentixafor, PET/CT, AVS, Adrenal nodules

## Abstract

**Background:**

Subtype diagnosis of primary aldosteronism (PA) is used to determine treatment, and the potential utility of ^68^Ga-pentixafor PET/CT for investigation of PA has long been recognized. The study aimed to evaluate the clinical value of ^68^Ga-pentixafor PET/CT in the diagnosis and prognosis of patients with bilateral lesions identified by CT.

**Methods:**

In total, 25 patients with PA and bilateral lesions on CT were retrospectively evaluated. All patients underwent ^68^Ga-Pentixafor PET/CT and adrenal vein sampling. The analysis focused on establishing the relationship between bilateral adrenal lesions SUVmax and the ratio of bilateral adrenal lesions SUVmax (CON) and clinical diagnosis, treatment outcomes, and KCNJ5 gene status.

**Results:**

The concordance rate between ^68^Ga-Pentixafor PET/CT and adrenal venous sampling was 65.2% (15/23). The lateralization results of ^68^Ga-pentixafor PET/CT supported the clinical decisions of 20 patients with PA, 90% of whom showed effectiveness in treatment. The SUVmax on the dominant side of the surgically treated patients was higher than that of patients treated with drugs. The SUVmax of the KCNJ5 mutant group was higher than that of the KCNJ5 wild group, and ^68^Ga-Pentixafor uptake was correlated with KCNJ5 gene status.

**Conclusions:**

^68^Ga-Pentixafor PET/CT proves beneficial for patients with PA with bilateral lesions on CT. The treatment is generally effective based on the results of PET lateralization. Simultaneously, a certain relationship exists between ^68^Ga-Pentixafor PET/CT and KCNJ5 gene status, warranting further analysis.

**Supplementary Information:**

The online version contains supplementary material available at 10.1186/s13550-024-01125-2.

## Introduction

Primary aldosteronism (PA) is marked by the autonomous hypersecretion of aldosterone and concurrent renin inhibition [1]. Unlike individuals with essential hypertension, patients with PA endure prolonged high aldosterone levels, resulting in heightened cardiovascular and renal damage [1, 2]. The lateralization of PA is used to determine treatment, adrenalectomy is the preferred choice for unilateral PA (UPA), and medication treatment is advised for bilateral PA (BPA). The postoperative clinical and biochemical remission rates for patients with PA far surpass those achieved through drug treatment [3, 4]. Hence, prompt diagnosis and treatment are imperative. Subtyping PA, a pivotal step in clinical decision-making, is crucial for identifying patients who stand to benefit most from surgery [5].

While adrenal vein sampling (AVS) stands as the current “gold standard” for typing diagnosis PA, it poses challenges such as operational difficulty and a lack of diagnostic consensus [6, 7]. For partial PA patients, the clinical and biochemical remission rates post-unilateral adrenalectomy based on CT decision are comparable to those based on AVS [8–10]. However, CT cannot accurately provide a subtyping PA for patients with bilateral lesions evident [9, 11]. Urgently needed are new typing methods except AVS for patients with PA with bilateral lesions observed on CT. A recent study by Wu X et al. revealed that AVS’s predictive accuracy for postoperative outcomes was inferior to that of metomidate (MTO) positron emission tomography (PET), MTO PET enables non-invasive detection of unilateral APAs [3], the main limitation of ^11^C-MTO is 20-min half-life, which limited clinical application.

The ligand PET imaging agent of CXC chemokine receptor type 4 (CXCR4), ^68^Ga-pentixafor, exhibits promising applications in tumors, heart disease, inflammation, and other diseases [12]. Heinze B et al. [13] first confirmed the close relationship between high ^68^Ga-pentixafor uptake in adrenocortical tissues and lesions with the expression of CXCR4 and aldosterone synthase (CYP11B2). Subsequent studies have indicated that ^68^Ga-pentixafor PET/CT holds significant clinical value in PA lateralization and prognosis. This study aims to meticulously analyze and evaluate the clinical utility of ^68^Ga-pentixafor PET/CT in the diagnosis and prognosis of patients with PA with bilateral lesions on CT, and comparing to AVS.

## Materials and methods

### Patients

From December 2021 to April 2023, we retrospectively included 25 patients with PA presenting bilateral adrenal lesions on CT, encompassing two patients with subclinical Cushing’s syndrome (SCS) (Patients 8 and 10). These patients were referred to us by clinic and bilateral adrenal lesions were identified by CT. All patients were diagnosed with PA by clinical endocrinologists following the guidelines of the Endocrine Society [5], and all participants completed the 1 mg dexamethasone inhibition test to determine whether they had complicated Cushing’s syndrome. Both AVS and ^68^Ga-pentixafor PET/CT were conducted, and the period between the two diagnostic tests was less than 2 weeks. Lesions were defined as adrenal nodules or thickenings with a transverse diameter ≥ 7 mm [3, 14]. All clinical data are obtained by a review of medical records. The study was approved by the Ethical Committee of our hospital and written informed consent was obtained from all patients.

### ^68^Ga-pentixafor uptake mechanism and synthesis

CXCR4 is highly expressed in aldosterone producing tissues, such as APA, and is closely related to CYP11B2, which is a crucial enzyme in the aldosterone synthesis. ^68^Ga-pentixafor is a radiolabeled CXCR4 ligand, visualization of ^68^Ga-pentixafor PET/CT revealed high radioactive uptake in APA and evaluated CXCR4 expression in APA [13].

The ^68^Ge/^68^Ga generator was subjected to a wash with hydrochloric acid, after which the ^68^Ga solution was obtained for radioactive labelling precursor pentixafor. The product, ^68^Ga-pentixafor, is a colourless solution with a pH of 5–8 and a radiochemical purity of greater than 99%.

### Image acquisition and analyses

Images were collected using Philips Gemini TF 64 PET/CT imaging equipment. No special preparation was required for the examinees. The imaging agent ^68^Ga-pentixafor 111–185 MBq (3–5 mCi) was injected into the median cubital vein, and images of the adrenal region were captured at 10 min and 40 min after the injection. The CT images were obtained with parameters set at a voltage of 120 keV, a current of 100 mA, a matrix of 512 × 512, and a layer thickness of 2 mm. Subsequently, PET images were acquired with acquisition parameters of 120 keV, a current of 100 mA, and a layer thickness of 3–5 mm. Maximum intensity projection images and PET/CT fusion images were generated using computer iterative reconstruction and attenuation correction.

Analysis of ^68^Ga-pentixafor PET/CT was performed using Lifex 6.20 software. The region of interest for adrenal lesions was manually delineated layer by layer in 2D mode, recording the SUVmax of bilateral lesions and the ratio of bilateral lesions SUVmax (CON). Lateralization was defined based on SUVmax differences > 25% on both sides [3, 15].

### Clinical management and prognosis evaluation

All patients underwent AVS firstly without adrenocorticotropic hormone (ACTH) stimulation. One patient encountered cannulation failure, subsequently undergoing successful AVS with a loading dose of ACTH (250 µg) stimulation. In total, 23 patients were successfully treated with AVS, while specific AVS data were unavailable for 2 patients. The criteria for successful cannulation and result interpretation were as follows: a selectivity index of ≥ 2 in the baseline state or ≥ 3 in the ACTH stimulation state indicated successful cannulation. A lateralization index of ≥ 4 or 2–4 combined with contralateral suppression was recorded as UPA. Contralateral suppression was defined when the contralateral suppression index was < 1, signifying that the aldosterone/cortisol ratio in the contralateral adrenal vein was lower than that in the inferior vena cava [16].

The aim of clinical decision-making is to attain optimal outcome, once ^68^Ga-pentixafor PET/CT or AVS support lateralization, surgery was recommended. Treatment decisions were made by endocrinologists and urologists based on clinical and ^68^Ga-pentixafor PET/CT data, and the patient’s willingness is also taken into consideration. Among the patients, 16 opted for surgical treatment, while 9 chose drug treatment. PASO guideline consensus was recommended for UPA with adrenalectomy, but we also assess patients with medication treatment for at least six months in compliance with the same consensus, in order to enable a clear comparison between surgery and medical therapy. Following the consensus [17, 18], preliminary therapeutic effects were evaluated at least six months post-treatment, classifying outcomes as effective (remission, improvement) or ineffective (persistence) based on clinical and biochemical aspects.

### Statistical analyses

Descriptive statistics were employed to characterize patient features. Continuous variables are expressed as mean ± SD or M (P25, P75), while categorical variables are presented as n (%). Fisher exact test and Mann–Whitney U test were utilized to compare the differences of clinical and semiquantitative variables (SUVmax, CON). Spearman correlation coefficient was calculated to assess the correlation between parameters. A significance level of *P* < 0.05 was applied for statistical significance. Data analysis was conducted using IBM SPSS Statistics 26.0 statistical software.

## Results

### Patient characteristics

This study included 25 patients with PA presenting bilateral nodules on CT, comprising 11 males and 14 females, with an average age of 52.8 ± 9.9 years. Within the operation group, characterized by older age, longer hypertension duration, lower blood pressure, higher aldosterone, and lower renin levels, and there were 5 patients with refractory hypertension. However, these differences were not statistically significant when compared with the drug treatment group (*P* > 0.05). Table 1 and Supplementary Table 1 provide an overview of the patients’ basic characteristics.

**Table 1:** Basic characteristics of patients

Characteristic	Total (*n* = 25)	Surgery *(*n* = 16)	Medications (*n* = 9)	*P* value
Age(year)	52.8 ± 9.9	53.4 ± 10.3	51.9 ± 9.7	0.890
Sex, Male, *n* (%)	11 (44%)	7 (43.8%)	4 (44.4%)	0.973
Hypertension History(year)	10.0 (2.0, 13.0)	10.0 (2.5, 13.0)	5.0 (1.3, 12.5)	0.559
Refractory hypertension, *n* (%)	5 (20%)	5 (31.3%)	0 (0)	0.363
Systolic BP (mmHg)	146.0 (130.5, 157.5)	141.5 (131.0, 151.0)	153.0 (130.0, 162.5)	0.357
Diastolic BP (mmHg)	91.2 ± 13.1	90.7 ± 11.6	92.1 ± 16.3	1.000
Hypokalemia history(year)	0.2 (0.1, 2.3)	0.2 (0.1, 2.1)	0.3 (0.1, 2.5)	0.677
Serum Potassium (mmol/L)	3.7 ± 0.5	3.8 ± 0.4	3.5 ± 0.6	0.152
PAC (ng/dL)	24.2 (19.5, 41.2)	26.8 (19.8, 46.8)	21.7 (15.8, 32.2)	0.108
PRA (ng/mL/h)	0.1(0.1, 0.4)	0.1 (0.1, 0.4)	0.2 (0.1, 0.4)	0.760
ARR ([ng/dL]/[ng/mL/h])	188.5 (57.6, 530.0)	220.6 (65.6, 662.5)	107.8 (52.9, 270.0)	0.419
positive CCT, *n* (%)	24 (96%)	16 (100%)	8 (88.9%)	0.667
positive SSIT, *n* (%)	23 (100%)	14 (100%)	9 (100%)	0.742

* Surgery group include a patient with superselective adrenal arterial embolization. Hypokalemia history based on 21 patients with hypokalemia. BP: blood pressure; PAC, plasma aldosterone concentration; PRA, plasma renin activity; ARR: plasma aldosterone renin ratio; CCT: captopril challenge test; SSIT: seated saline infusion test

### Comparison of subtyping PA results between ^68^Ga-pentixafor PET and AVS

The CON based on 10 min SUVmax and 40 min SUVmax was 1.53 (1.29, 2.85) and 1.38 (1.19, 3.64), respectively. No significant correlation was observed between the lateralization index of AVS [8.26 (1.66, 14.98); *P* > 0.05]. Table 2 and Supplementary Table 2 detail the diagnosis, treatment, and prognosis of the patients.

**Table 2:** Lateralization, treatment and prognosis of patients

	PET-SUVmax				AVS			Treatment	KCNJ5	Biochemical	Clinical
NO.	CON*	Side*	CON#	Side#	LI	CSI	Side			Outcome	Outcome
1	1.69	R	1.38	R	13.27	0.04	R	Operation (R)	mutation	remission	improvement
2	1.02	Bξ	1.14	Bξ	9.92	0.62	Lε	Operation (L)	wild	remission	improvement
3	3.52	R	3.82	R	13.99	0.14	R	Operation (R)	mutation	remission	remission
4	1.08	Bξ	1.04	Bξ	6.81	0.44	L	Medication	NA	remission	improvement
5	1.32	Lξ	1.27	Lξ	1.45	1.32	B	Operation (L)	mutation	remission	improvement
6	2.12	Rξ	2.64	Rξ	1.73	1.65	B	Operation (R)	mutation	improvement	improvement
7	1.53	R	1.44	R	15.97	0.30	R	Operation (R)	mutation	remission	remission
8	3.17	Rξ	4.28	Rξ	1.96	1.49	B	Operation (R)	mutation	remission	remission
9	1.30	R	1.23	Bξ	8.42	0.55	R	Operation (R)	mutation	remission	remission
10	2.13	R	2.37	R	8.26	0.09	R	Operation (R)	wild	remission	improvement
11	2.00	R	2.59	R	16.43	0.07	R	Medication	NA	improvement	improvement
12	1.09	B	1.20	B	1.45	0.41	B	Medication	NA	improvement	improvement
13	4.18	R	8.56	R	23.24	0.34	R	Operation (R)	mutation	remission	remission
14	1.35	Rξ	1.29	Rξ	1.59	1.03	B	Medication	NA	improvement	improvement
15	1.37	Rξ	1.12	B	1.26	1.40	B	Medication	NA	improvement	improvement
16	1.09	B	1.06	B	NA	NA	B	Medication	NA	improvement	improvement
17	3.31	R	5.17	R	NA	NA	R	SAAE(R)	NA	persistence	persistence
18	1.01	Bξ	1.07	Bξ	18.42	0.11	R	Operation (R)	wild	remission	remission
19	1.27	R	1.19	B	NA	NA	NA	Medication	NA	remission	remission
20	5.09	L	4.84	L	NA	NA	NA	Operation (L)	mutation	remission	remission
21	2.54	L	3.45	L	84.15	0.05	L	Operation (L)	NA	remission	improvement
22	3.15	R	5.28	R	4.85	0.70	R	Operation (R)	NA	remission	improvement
23	1.45	Lξ	1.29	Lξ	1.29	1.12	B	Medication	NA	improvement	improvement
24	1.16	B	1.08	B	1.74	0.32	B	Medication	NA	improvement	persistence
25	2.17	L	1.59	L	8.42	0.21	L	Operation (L)	wild	remission	improvement

ξ Different from AVS lateralization. ε Corticotropin stimulation in adrenal vein sampling. * 10 min; # 40 min. AVS: adrenal vein sampling; LI: lateralization index; CSI: contralateral suppression index; NA, not available; SAAE: superselective adrenal arterial embolization

**Fig. 1:**
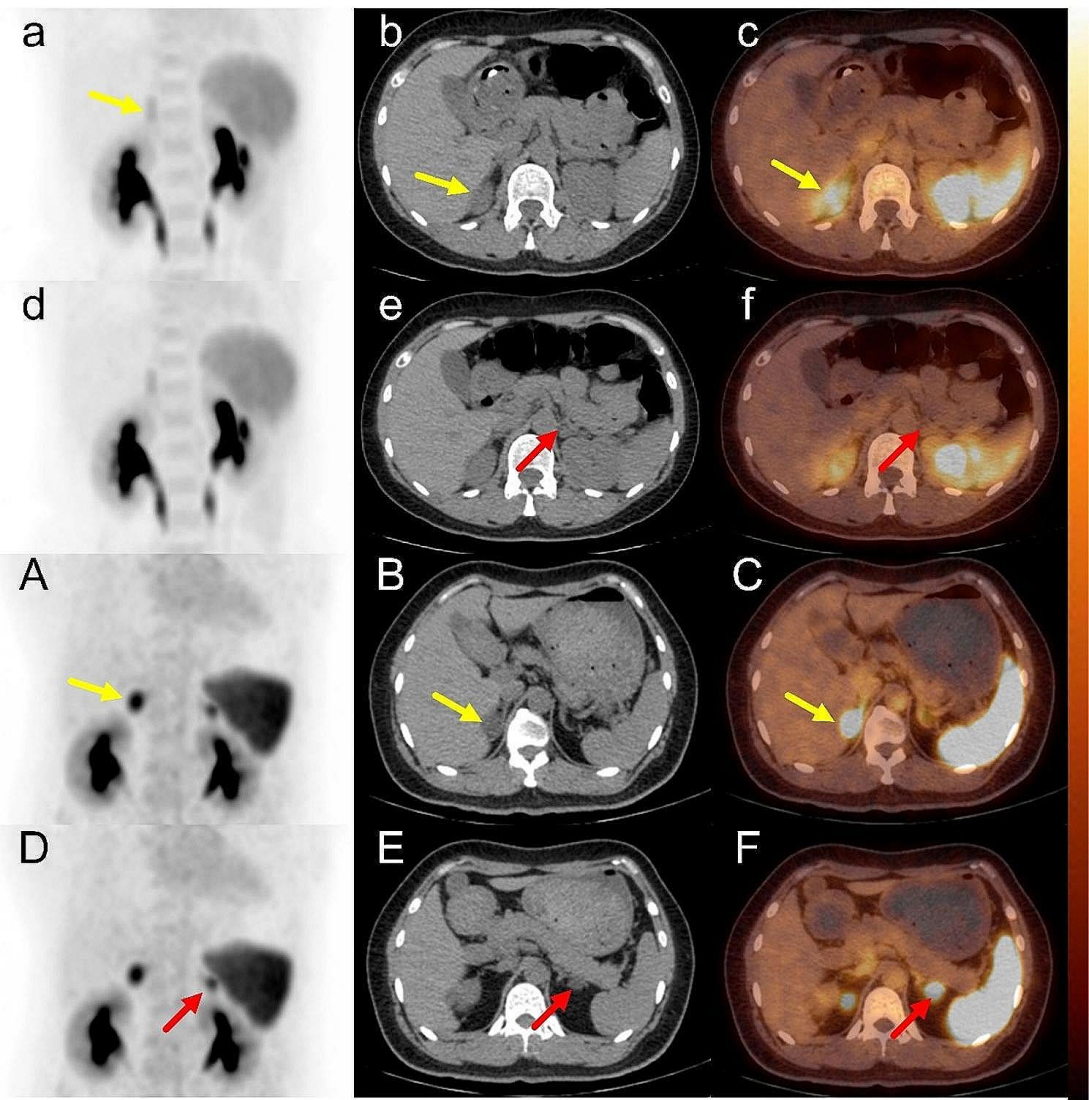
The 10-min ^68^Ga-pentixafor PET lateralization result **(a-f)** of patient 9 was consistent with AVS, both of which were UPA (right). **a-c**: right adrenal gland, **d-f**: left adrenal gland; right SUVmax = 5.41 (yellow arrow), left SUVmax = 4.17 (red arrow), CON = 1.30. 40 min ^68^Ga-pentixafor PET lateralization result **(A-F)** of patient 15 was consistent with AVS, which was BPA. **A-C**: right adrenal gland, **D-F**: left adrenal gland; right SUVmax = 12.43 (yellow arrow), left SUVmax = 11.13 (red arrow), and CON = 1.12.

**Fig. 2:**
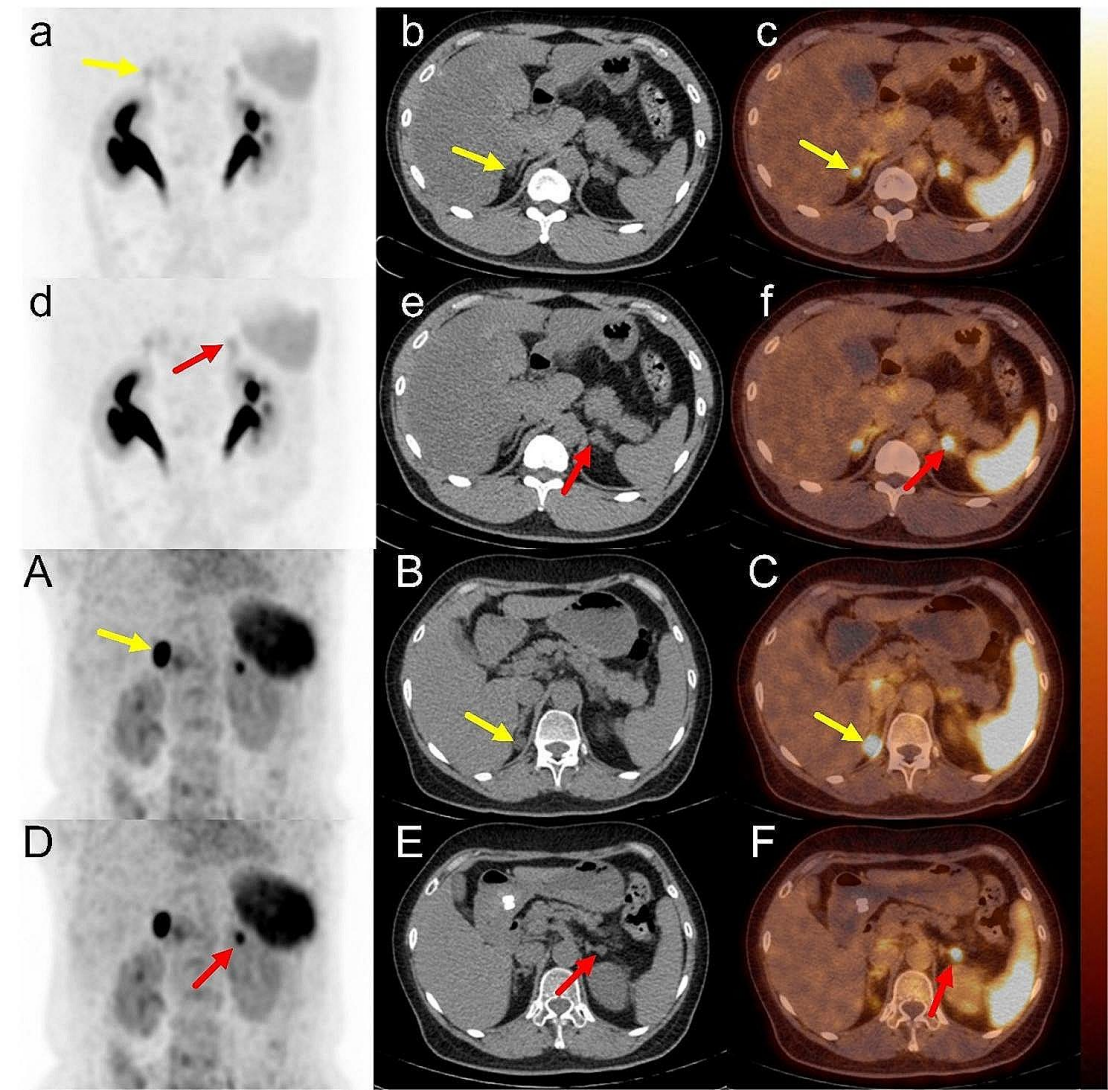
The 10-min ^68^Ga-pentixafor PET lateralization result **(a-f)** of patient 18 showed BPA, and AVS indicated UPA (right, LI = 18.42). **a-c**: right adrenal gland, SUVmax = 4.90 (yellow arrow); **d-f**: left adrenal gland, SUVmax = 4.87 (red arrow); CON = 1.01. 10 min ^68^Ga-pentixafor PET lateralization result **(A-F)** of patient 6 showed UPA (right), and AVS indicated BPA (LI = 1.73). **A-C**: right adrenal gland, SUVmax = 20.20 (yellow arrow); **D-F**: left adrenal gland, SUVmax = 9.55 (red arrow); and CON = 2.12.

The agreement between lateralization results based on ^68^Ga-pentixafor PET (10 min and 40 min) and AVS was 65.2% (15/23). Except for patients 9 and 15, whose PET lateralization results did not align at 10 min and 40 min, there were no differences in PET lateralization results for the other patients at the two-time points. The lateralization results of 10 min PET for patient 9 and 40 min PET for patient 15 were consistent with those of AVS (Fig. 1 and Supplementary Fig. 1). Patients 2, 4, and 18 exhibited BPA in dual-point PET (10 and 40 min), while AVS indicated UPA (Group 1). On the other hand, patients 5, 6, 8, 14, and 23 showed UPA in imaging, while AVS suggested BPA (Group 2). Group 2 exhibited relatively high ^68^Ga-pentixafor uptake compared to Group 1 (Fig. 2, Supplementary Fig. 2, and Supplementary Table 3).

**Fig. 3:**
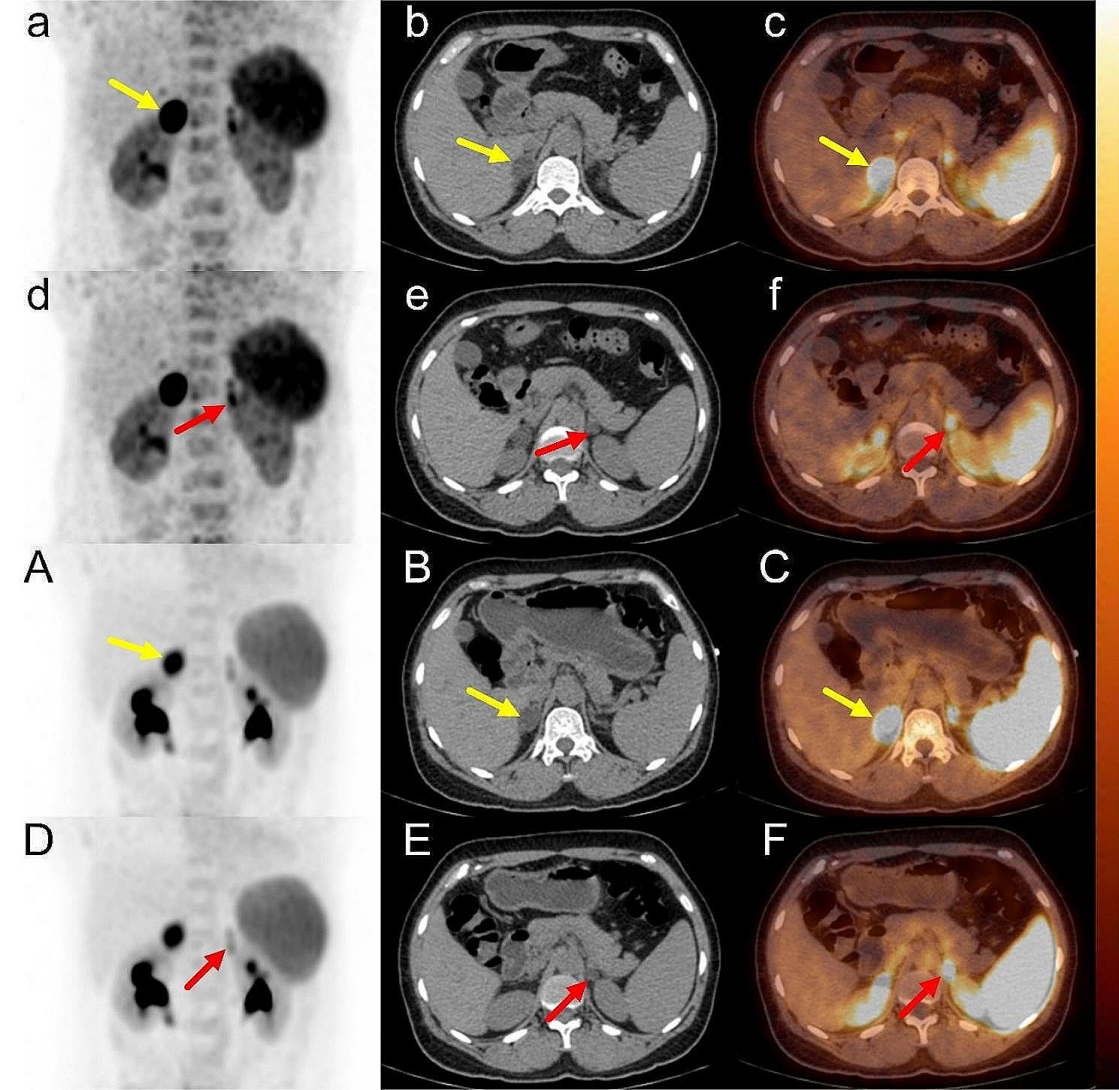
Patient 8 was complicated with SCS. The level of cortisol after 1 mg dexamethasone inhibition test was 115.06 nmol/L, and 24 h-free urinary cortisol was 523.28 nmol/L, with no typical sign of Cushing syndrome. Both 10-min and 40-min ^68^Ga-pentixafor PET indicated UPA (right) (10 min: a-f; 40 min: **A-F**), while AVS showed BPA (LI = 1.96). **a-c**: right adrenal gland, SUVmax = 25.93 (yellow arrow); **d-f**: left adrenal gland, SUVmax = 8.18 (red arrow); CON = 3.17. **A-C**: right adrenal gland, SUVmax = 30.39 (yellow arrow); **D–F**: left adrenal gland, SUVmax = 7.10 (red arrow); CON = 4.28.

Patient 8 presented with PA complicated by SCS. The right nodule displayed significantly increased radioactivity uptake (10 min SUVmax = 25.93) that continued to rise with time (40 min SUVmax = 30.39), significantly surpassing that of the contralateral adrenal nodule (10 / 40 min CON: 3.17 / 4.28). However, AVS indicated BPA (LI = 1.96). After comprehensive consideration, right adrenalectomy was performed and clinical and biochemical remission was achieved (Fig. 3).

### Prognosis evaluation of ^68^Ga-pentixafor PET

Nine patients opted for drug therapy; among them, patient 4 suspended operation due to right femoral artery thrombosis, patient 11 refused operation, and patient 19, whose AVS failed, chose drug treatment based on PET results. The remaining six patients decided on drug treatment according to AVS results. Except for patient 24, who showed no clinical remission and only achieved biochemical improvement, the other patients experienced effective treatment. Among those treated with drugs, 77.8% achieved clinical and biochemical improvement, with only two patients achieving biochemical remission and one patient achieving clinical remission (Supplementary Table 4).

Sixteen patients opted for surgery, with 15 undergoing unilateral adrenalectomy. Patients 5 and 6 considered the possibility of bilateral aldosteronoma based on clinical and PET results, selecting unilateral adrenalectomy based on PET findings. Patient 8, complicated with SCS, underwent right adrenalectomy based on PET results as AVS results showed BPA(LI = 1.96). Patient 20, whose AVS failed, chose adrenalectomy based on PET results. The other 11 patients underwent surgery based on AVS results. All patients who underwent unilateral adrenalectomy achieved biochemical and clinical effective treatment. The results of PET and AVS in patients with clinical improvement were not significantly different from those with clinical remission (*P* > 0.05, Supplementary Table 4).

^68^Ga-pentixafor PET results supported 80% of clinical decisions, including 14 cases of surgery and 6 cases of drug treatment, all of which were effective treatment (clinical and biochemical are remission or improvement). The SUVmax of the dominant side in surgical patients was higher than that in patients treated with drugs (10 min: 13.63 ± 6.20 vs. 7.43 ± 3.75, *P* = 0.009; 40 min: 12.59 ± 7.85 vs. 6.67 ± 3.78, *P* = 0.031). The biochemical and clinical remission rates were higher for surgical patients compared to those treated with drugs, with statistically significant differences in biochemical remission rates (22.2% vs. 93.3%, *P* = 0.001; 11.1% vs. 40%, *P* = 0.191).

Patient 17 was transferred from the Department of cardiovascular medicine and performed superselective adrenal artery embolization after ^68^Ga-pentixafor PET/CT. ^68^Ga-pentixafor PET/CT revealed a significant decrease in radioactivity uptake in the right nodules on the second day after the operation (10 min SUVmax = 9.97 < 17.90). On the second day after the operation, serum potassium and ARR were 3.5 mmol/L and 35.19 [ng/dL]/[ng/mL/h], respectively. Three months post-operation, PET showed a significant increase in uptake in the right nodule (10 min SUVmax = 26.02). The patient’s blood pressure was 139/91 mmHg, and the serum potassium level was 3.3 mmol/L, with an ARR of 206.70 [ng/dL]/[ng/mL/h], and resulting in clinical and biochemical persistence (Supplementary Fig. 3 and Supplementary Fig. 4). However, patient 17 was not willing to undergo secondary embolization and right adrenalectomy, opting for later drug treatment.

### Correlation of ^68^Ga-pentixafor PET with KCJN5

Out of the 15 patients who underwent unilateral adrenalectomy, 13 underwent KCNJ5 detection, with 9 exhibiting KCNJ5 mutations and 4 showing the KCNJ5 wild type. The SUVmax of the KCNJ5 mutant group was higher than that of the KCNJ5 wild group (10 min: 15.31 ± 6.16 vs. 7.9 ± 2.49, *P* = 0.026; 40 min: 14.20 ± 8.49 vs. 6.78 ± 1.74, *P* = 0.117).

## Discussion

In this study, the lateralization results of ^68^Ga-pentixafor PET/CT supported the clinical decision-making of 20 patients with PA. Except for patient 17 who underwent superselective adrenal artery embolization and patient 24 treated with drugs, all patients exhibited clinical and biochemical effectiveness. The inconsistency rate between ^68^Ga-pentixafor PET/CT and AVS in PA typing diagnosis was 34.8% (8/23). Among them, patients 4, 5, 6, and 8 were treated according to PET results, with 75% (patients 4, 5, 8) achieving biochemical remission, and patient 6 achieving biochemical improvement, all while experiencing improved clinical outcomes. For patients with PA with bilateral nodules, ^68^Ga-pentixafor PET/CT accurately lateralized and guided the clinical treatment.

While CT is a crucial tool for diagnosing adrenal lesions in patients with PA, it cannot replace AVS in the lateralization of PA, especially for patients with CT showing bilateral lesions [11, 19, 20]. Aono D et al. [9] found a lateralization inconsistency rate between CT and AVS of 39% (74/191) in patients with bilateral lesions on adrenal CT. Despite this, CT is routinely performed before AVS, primarily to rule out the possibility of large adrenal masses and secondly to observe the structure of the adrenal vein, reducing the risk of AVS failure [19]. Importantly, after AVS failure, CT can still provide partial profile information [20]. However, the lateralization consistency rate between ^68^Ga-pentixafor PET and AVS found in this study was 65.2%, surpassing that of CT in patients with PA with bilateral lesions [9, 20]. In future clinical practice, it may be possible to replace CT with ^68^Ga-pentixafor PET/CT, providing more functional information alongside anatomical details.

AVS failure can be attributed to adrenal anatomical variation, inexperienced operators, and fluctuating cortisol levels [21]. In clinical practice, AVS failure implies the loss of the opportunity for surgery, often leading to drug therapy with relatively suboptimal therapeutic outcomes [22]. Nuclear medicine functional imaging plays a crucial role in the lateralization and prognosis of PA. The accuracy of ^11^C-MTO PET in predicting biochemical and clinical remission after adrenalectomy surpasses that of AVS, making it a valuable diagnostic tool [3]. Similarly, ^68^Ga-pentixafor PET/CT demonstrates high accuracy in PA localization and holds promise as a potential non-invasive substitute for AVS [16, 23, 24]. In this study, patients 19 and 20, who experienced AVS failure, achieved clinical and biochemical remission through medicine and surgery, respectively, based on PET results. This highlights the potential of avoiding repeated AVS while expanding the operable target population in a non-invasive manner.

KCNJ5 mutation is related to the pathogenesis of sporadic and familial PA, and familial hyperaldosteronism type III could be excluded by KCNJ5 gene detection [25, 26]. KCNJ5 mutation is also associated with changes in cardiac structure, function and metabolic disorders in patients with PA [27, 28]. In a word, KCNJ5 is a pivotal gene in PA, and with over 65% of the PA population in Southeast Asia associated with KCNJ5 mutation [29]. Study have shown that patients with KCNJ5 mutations exhibit higher uptake in imaging agents such as ^11^C-MTO and ^131^I-NP-59, potentially linked to CYP11B2 overexpression caused by KCNJ5 mutations [18]. In this study, the relationship between KCNJ5 and ^68^Ga-pentixafor uptake was investigated for the first time, revealing that the KCNJ5 mutant group had higher SUVmax than the KCNJ5 wild group. KCNJ5 mutations alter depolarization levels by mediating inward rectifier potassium channels, leading to increased calcium influx, overexpression of CYP11B2, and heightened aldosterone secretion [29, 30]. Given the close association between CXCR4, CYP11B2 expression and KCNJ5 gene [13, 24, 30], the increased ^68^Ga-pentixafor uptake may be linked to KCNJ5 gene, although it needs further evaluation with larger datasets.

Interestingly, two patients with PA (patient 8, 10) complicated by SCS were included in the study. Both exhibited relatively large main lesions and high ^68^Ga-pentixafor uptake, especially in patient 8, where the SUVmax of the right lesion significantly exceeded that of the contralateral side (10 min CON = 4.28). While PET suggested right UPA (10 min CON = 4.28), AVS results showed bilateral disease (LI = 1.96). AVS, relying on cortisol to correct aldosterone levels, may produce false-negative results in the presence of SCS-associated spontaneous cortisol secretion [31], as observed in patient 8. Both patients underwent right adrenalectomy and achieved biochemical and clinical effective treatment. Patient 8 exhibiting KCNJ5 mutation and higher ^68^Ga-pentixafor uptake than patient 10 (KCNJ5 wild). Despite the challenges posed by SCS, ^68^Ga-pentixafor PET/CT demonstrated its potential to reflect the lateralization and status of KCNJ5 in patients with PA with SCS.

This study has several limitations. First, the relatively small sample size might not fully capture the potential value of ^68^Ga-pentixafor PET/CT in patients with PA and bilateral lesions. Second, the limited number of patients experiencing treatment ineffectiveness, whether through surgery or drug treatment, hindered an in-depth analysis of the differences in treatment outcomes related to ^68^Ga-pentixafor uptake. Third, as the treatment of PA is primarily based on AVS results, evaluating the accuracy of ^68^Ga-pentixafor PET lateralization proves challenging, emphasizing the need for multicenter prospective study. In addition, the limited KCNJ5 results is difficult to reflect the relationship between KCNJ5 and ^68^Ga-pentixafor SUVmax, which requires more samples to further confirm the results.

## Conclusions

Our study demonstrates that ^68^Ga-pentixafor PET/CT can favor lateralization in patients with PA with bilateral lesions, aiding in reflecting KCNJ5 gene status and treatment outcomes. In instances of no AVS or AVS failure, ^68^Ga-pentixafor PET/CT emerges as a valuable tool for PA lateralization. However, these findings necessitate validation through prospective multicenter study to establish their robustness and reliability in clinical practice.

### Electronic supplementary material

Below is the link to the electronic supplementary material.

Supplementary Material 1

## Data Availability

The datasets generated or analyzed during the study are available from the corresponding author on reasonable request.

## Abbreviations

PET/CT: Pemission Tomography/Computed Tomography
PA: Primary Aldosteronism
CON: The Ratio of Bilateral Adrenal Lesions SUVmax
UPA: Unilateral PA
BPA: Bilateral PA
AVS: Adrenal Vein Sampling
MTO: Metomidate
PET: Positron Emission Tomography
CXCR4: CXC Chemokine Receptor Type 4
CYP11B2: Aldosterone Synthase
SCS: Subclinical Cushing’s Syndrome
ACTH: Adrenocorticotropic Hormone
