# Supplementary material for: Clinical value of 68Ga-pentixafor PET/CT in patients with primary aldosteronism and bilateral lesions: preliminary results of a single-centre study
Source: EJNMMI Res. 2024 Jul 4;14:61. doi: 10.1186/s13550-024-01125-2 (PMC11224210; doi:10.1186/s13550-024-01125-2)
Supplement: Supplementary file 1 — Supplementary Material 1 [file 13550_2024_1125_MOESM1_ESM.docx]

Supplementary table 1 Basic characteristics of patients

| No. | Age(year) | Sex | Hypertension  History (year) | Refractory  hypertension | Systolic BP  (mmHg) | Diastolic BP  (mmHg) | Hypokalemia  History (year) | Serum Potassium  (mmol/L) | PAC  (ng/dL) | PRA  (ng/mL/h) | ARR  ([ng/dL]/[ng/mL/h]) | Positive  CTT | positive  SSIT |
| --- | --- | --- | --- | --- | --- | --- | --- | --- | --- | --- | --- | --- | --- |
| 1 | 47 | F | 10 | No | 131 | 89 | 4 | 4.0 | 26.7 | 0.37 | 72.76 | Yes | Yes |
| 2 | 64 | M | 13 | Yes | 151 | 91 | 0.1 | 4.0 | 15.3 | 0.33 | 45.94 | Yes | Yes |
| 3 | 65 | M | 15 | Yes | 132 | 77 | 0.1 | 4.3 | 18.9 | 0.13 | 145.17 | Yes | Yes |
| 4 | 56 | M | 0.3 | No | 167 | 89 | 0.3 | 4.2 | 23.1 | 0.21 | 107.87 | Yes | Yes |
| 5 | 36 | F | 10 | No | 169 | 100 | 3 | 3.9 | 34.8 | 0.14 | 252.66 | Yes | / |
| 6 | 56 | F | 13 | No | 146 | 109 | 2.5 | 4.0 | 55.6 | 0.58 | 96.07 | Yes | Yes |
| 7 | 50 | F | 2 | No | 128 | 75 | 1 | 3.4 | 10.5 | 0.46 | 22.67 | Yes | Yes |
| 8 | 48 | F | 11 | Yes | 128 | 93 | 0.2 | 4.4 | 32.5 | 0.07 | 485.21 | Yes | Yes |
| 9 | 35 | F | 0.1 | No | 137 | 108 | 0 | 3.0 | 23.0 | 0.71 | 32.55 | Yes | Yes |
| 10 | 66 | M | 20 | No | 167 | 80 | 0.2 | 3.9 | 55.3 | 0.05 | 1208.62 | Yes | Yes |
| 11 | 39 | F | 5 | No | 157 | 100 | 3 | 3.5 | 20.1 | 0.09 | 226.66 | Yes | Yes |
| 12 | 45 | M | 10 | No | 171 | 121 | 0.1 | 3.0 | 24.2 | 0.46 | 52.62 | Yes | Yes |
| 13 | 61 | F | 2 | No | 149 | 75 | 0.5 | 4.5 | 26.1 | 0.04 | 691.65 | Yes | Yes |
| 14 | 57 | M | 1 | No | 141 | 95 | 10 | 4.4 | 10.2 | 0.05 | 191.36 | Yes | Yes |
| 15 | 35 | F | 3.5 | No | 130 | 75 | 2 | 2.6 | 40.1 | 0.13 | 312.54 | Yes | Yes |
| 16 | 69 | F | 31 | Yes | 197 | 89 | 0 | 3.5 | 13.8 | 0.22 | 63.19 | Yes | / |
| 17 | 58 | F | 20 | No | 153 | 90 | 0 | 3.5 | 21.7 | 0.35 | 62.17 | Yes | Yes |
| 18 | 57 | M | 0.1 | No | 151 | 109 | 0.1 | 3.6 | 26.9 | 0.02 | 1140.56 | Yes | Yes |
| 19 | 58 | F | 10 | No | 121 | 66 | 0.5 | 2.7 | 46.5 | 0.03 | 1643.00 | Yes | Yes |
| 20 | 42 | F | 4 | No | 135 | 82 | 0.1 | 3.8 | 42.3 | 0.07 | 574.85 | Yes | Yes |
| 21 | 50 | M | 12 | No | 150 | 98 | 0 | 3.5 | 48.3 | 0.06 | 775.73 | Yes | Yes |
| 22 | 52 | M | 4 | No | 131 | 91 | 4 | 3.3 | 49.5 | 0.12 | 416.43 | Yes | Yes |
| 23 | 62 | F | 1.5 | No | 130 | 87 | 0 | 3.8 | 11.4 | 0.21 | 53.12 | No | Yes |
| 24 | 57 | M | 15 | No | 158 | 106 | 0.2 | 3.4 | 21.1 | 1.02 | 20.60 | Yes | Yes |
| 25 | 57 | M | 7 | Yes | 124 | 85 | 0.1 | 3.7 | 22.5 | 0.12 | 188.54 | Yes | Yes |

BP: blood pressure; PAC, plasma aldosterone concentration; PRA, plasma renin activity; ARR: plasma aldosterone renin ratio; CCT: captopril challenge test; SSIT: seated saline infusion test.

Supplementary table 2 ^68^Ga-pentixafor PET/CT performance of patients

| No. | Left |  | |  |  | Right |  |  | |  | |
| --- | --- | --- | --- | --- | --- | --- | --- | --- | --- | --- | --- |
|  | Size (cm) | 10min SUVmax | 40min SUVmax | | ▲SUVmax | Size (cm) | 10min SUVmax | | 40min SUVmax | | ▲SUVmax |
| 1 | 0.8 | 5.6 | 5.83 | | -0.23 | 1.2 | 9.46 | | 8.06 | | 1.4 |
| 2 | 2.5 | 7.3 | 6.5 | | 0.80 | 1.9 | 7.43 | | 5.7 | | 1.73 |
| 3 | 0.7 | 4.03 | 2.43 | | 1.60 | 1.6 | 14.2 | | 9.28 | | 4.92 |
| 4 | 0.7 | 7.07 | 5.42 | | 1.65 | 0.8 | 6.57 | | 5.23 | | 1.34 |
| 5 | 1.7 | 9.96 | 10.62 | | -0.66 | 1.1 | 7.54 | | 8.36 | | -0.82 |
| 6 | 1.0 | 9.55 | 7.87 | | 1.68 | 1.5 | 20.2 | | 20.79 | | -0.59 |
| 7 | 1.4 | 8.52 | 5.98 | | 2.54 | 1.3 | 13.06 | | 8.61 | | 4.45 |
| 8 | 1.0 | 8.18 | 7.1 | | 1.08 | 2.4 | 25.93 | | 30.39 | | -4.46 |
| 9 | 0.7 | 4.17 | 3.46 | | 0.71 | 1.0 | 5.41 | | 4.24 | | 1.17 |
| 10 | 1.5 | 5.08 | 3.81 | | 1.27 | 2.5 | 10.84 | | 9.04 | | 1.8 |
| 11 | 0.7 | 6.76 | 4.97 | | 1.79 | 1.2 | 13.49 | | 12.86 | | 0.63 |
| 12 | 1.6 | 7.87 | 7.55 | | 0.32 | 1.9 | 7.2 | | 9.09 | | -1.89 |
| 13 | 1.5 | 4.61 | 2.75 | | 1.86 | 0.8 | 19.25 | | 23.54 | | -4.29 |
| 14 | 1.2 | 3.26 | 2.62 | | 0.64 | 0.7 | 4.41 | | 3.38 | | 1.03 |
| 15 | 1.3 | 10.36 | 11.13 | | -0.77 | 2.4 | 14.17 | | 12.43 | | 1.74 |
| 16 | 2.9 | 4.76 | 4.48 | | 0.28 | 1.0 | 5.2 | | 4.74 | | 0.46 |
| 17 | 0.7 | 5.4 | 3.5 | | 1.90 | 1.5 | 17.9 | | 18.1 | | 1.9 |
| 18 | 1.2 | 4.87 | 5.14 | | -0.27 | 1.0 | 4.90 | | 4.82 | | 0.08 |
| 19 | 1.5 | 3.94 | 3.7 | | 0.24 | 1.0 | 4.99 | | 4.41 | | 0.58 |
| 20 | 1.5 | 19.86 | 18.39 | | 1.47 | 0.9 | 3.9 | | 3.8 | | 0.1 |
| 21 | 1.9 | 20 | 17.73 | | 2.27 | 1.3 | 7.88 | | 5.14 | | 2.74 |
| 22 | 0.9 | 3.53 | 0.87 | | 2.66 | 1.2 | 11.12 | | 4.59 | | 6.53 |
| 23 | 1.5 | 5.21 | 3.73 | | 1.48 | 1.0 | 3.6 | | 2.89 | | 0.71 |
| 24 | 0.8 | 4.4 | 3.72 | | 0.68 | 1.0 | 5.12 | | 4.0 | | 1.12 |
| 25 | 3.0 | 8.7 | 6.71 | | 1.99 | 1.5 | 4.01 | | 4.22 | | -0.21 |

▲ The difference between two time points of SUVmax (10 min SUVmax-40 min SUVmax).

Supplementary table 3 Difference of patients with different subtype findings

|  | PET-AVS+（n=3） | PET+AVS-（n=5） |
| --- | --- | --- |
| 10 min-SUVmax (left) | 6.41±1.34 | 7.23±2.90 |
| 10 min-SUVmax (right) | 6.30±1.29 | 12.34±10.11 |
| 10 min-CON | 1.16±0.19 | 1.88±0.79 |
| 40 min-SUVmax (left) | 5.69±0.72 | 6.38±3.24 |
| 40 min-SUVmax (right) | 5.25±0.44 | 13.16±12.03 |
| 40 min-CON | 1.10±0.53 | 2.15±1.33 |
| LI | 11.72±6.01 | 1.60±0.26 |
| CSI | 0.39±0.26 | 1.45±0.29 |

AVS: adrenal vein sampling; LI: lateralization index; CSI: contralateral suppression index.

Supplementary table 4 Comparison of PET and AVS parameters between different outcome

|  | Dominant side | | Non-dominant side | |  |  |  |  |
| --- | --- | --- | --- | --- | --- | --- | --- | --- |
|  | 10 min-SUVmax | 40 min-SUVmax | 10 min-SUVmax | 40 min-SUVmax | 10 min-CON | 40 min-CON | LI | CSI |
| Medicine |  |  |  |  |  |  |  |  |
| Biochemical remission (n=2) | 7.07; 4.99 | 5.42; 4.41 | 6.57; 3.94 | 5.23; 3.70 | 1.08; 1.27 | 1.04; 1.19 | 0.44; / | 6.81; / |
| Biochemical improvement (n=7) | 8.12 ± 4.07 | 6.94 ± 4.13 | 5.81 ± 2.50 | 5.45 ± 3.32 | 1.39 ± 0.30 | 1.40 ± 0.53 | 1.52 (1.28, 5.41) | 0.72 (0.26, 1.50) |
| *P* value | NA | NA | NA | NA | NA | NA | NA | NA |
| Clinical remission (n=1) | 4.99 | 4.41 | 3.94 | 3.70 | 1.27 | 1.19 | / | / |
| Clinical improvement (n=7) | 8.40 ± 3.89 | 7.15 ± 3.99 | 6.12 ± 2.43 | 5.67 ± 3.24 | 1.38 ± 0.31 | 1.40 ± 0.53 | 1.52 (1.28, 9.22) | 0.74 (0.33, 1.50) |
| Clinical persistence (n=1) | 5.12 | 4.00 | 4.40 | 3.72 | 1.16 | 1.08 | 0.32 | 1.74 |
| *P* value | NA | NA | NA | NA | NA | NA | NA | NA |
| Operation |  |  |  |  |  |  |  |  |
| Biochemical remission (n=14) | 11.82 ± 6.31 | 10.07 ± 7.47 | 5.34 (4.02, 7.95) | 5.14 (3.72, 6.65) | 2.41 ± 1.25 | 1.98 (1.26, 4.42) | 9.92 (6.56, 17.20) | 0.30 (0.10, 0.66) |
| Biochemical improvement (n=1) | 20.20 | 20.79 | 9.55 | 7.87 | 2.12 | 2.64 | 1.73 | 1.65 |
| *P* value | NA | NA | NA | NA | NA | NA | NA | NA |
| Clinical remission (n=6) | 12.30 ± 8.99 | 6.71 (3.87, 21.39) | 8.15 ± 5.80 | 5.56 (3.72, 11.21) | 2.71 ± 1.69 | 2.86 (1.19, 5.77) | 13.60 ± 8.43* | 0.34 (0.21, 1.02) * |
| Clinical improvement (n=9) | 12.43 ± 4.73 | 10.28 ± 5.47 | 6.06 ± 2.10 | 5.00 ± 2.45 | 2.18 ± 0.81 | 2.55 ± 1.41 | 8.42 (3.29, 13.63) | 0.21 (0.07, 1.01) |
| *P* value | 0.556 | 0.556 | 0.556 | 0.680 | 0.814 | 0.814 | 0.641 | 0.286 |

* Includes 5 patients. LI: lateralization index; CSI: contralateral suppression index; NA, not available.


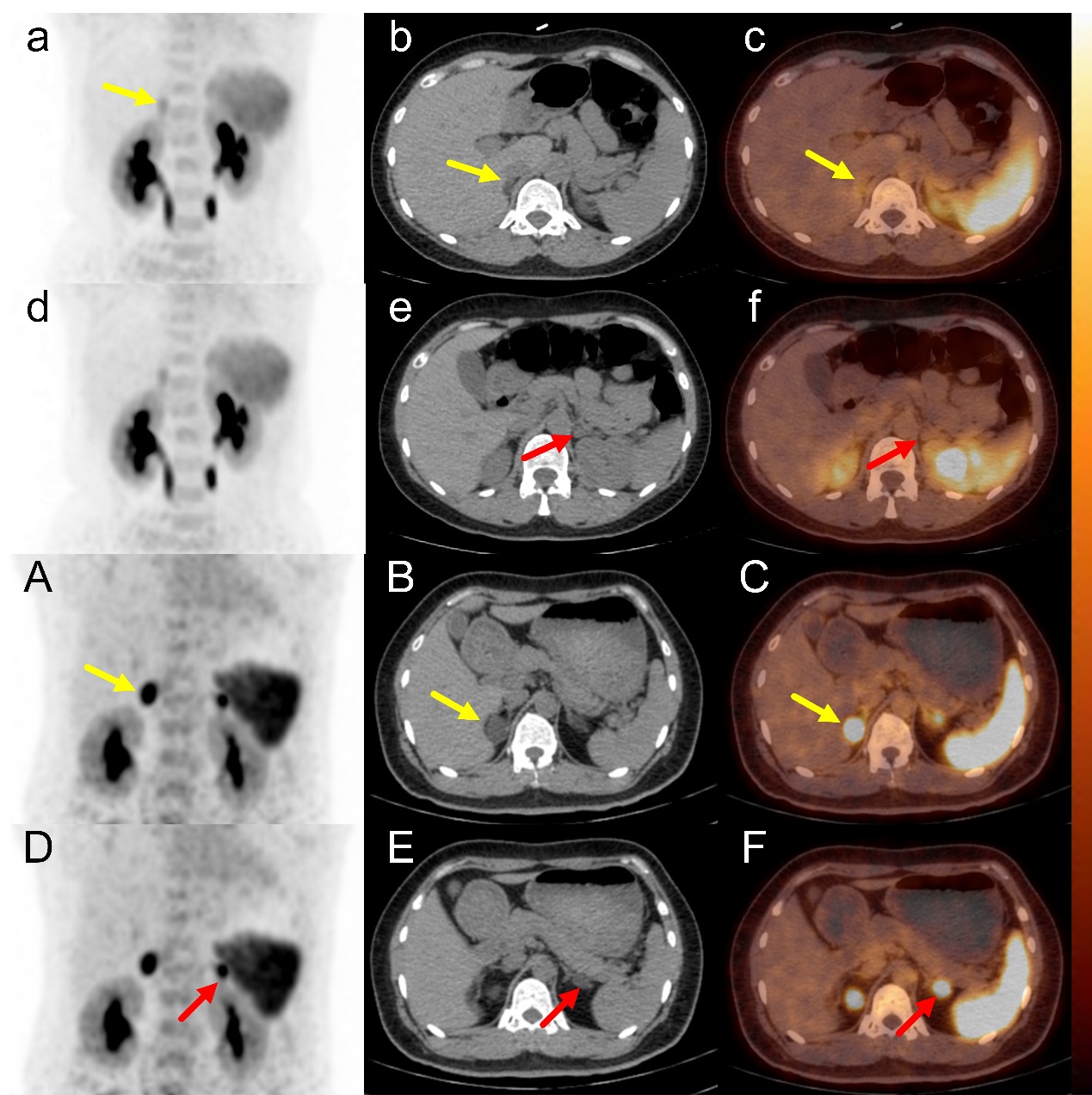


Supplementary figure 1 The 40-min ^68^Ga-pentixafor PET lateralization result (a-f) of patient 9 showed BPA, and AVS indicated UPA (right). a-c: right adrenal gland, SUVmax = 4.24 (yellow arrow); d-f: left adrenal gland, SUVmax = 3.46 (red arrow); CON = 1.23. 10 min ^68^Ga-pentixafor PET lateralization result (A-F) of patient 15 showed UPA (right), and AVS indicated BPA. A-C: right adrenal gland, SUVmax = 14.17 (yellow arrow); D-F: left adrenal gland, SUVmax = 10.36 (red arrow); and CON = 1.37.


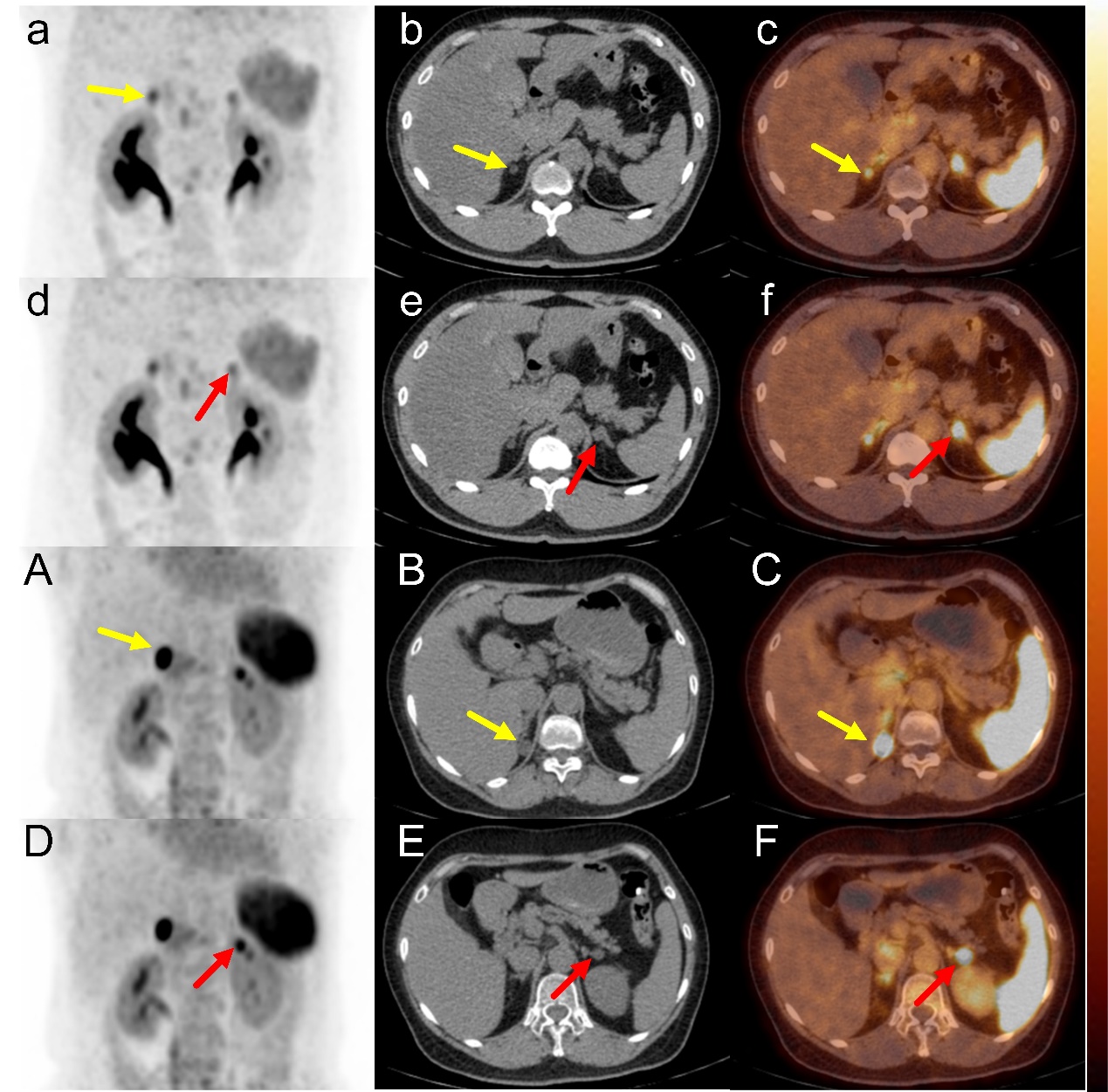


Supplementary figure 2 The 40-min ^68^Ga-pentixafor PET lateralization result (a-f) of patient 18 showed BPA, and AVS indicated UPA (right). a-c: right adrenal gland, SUVmax = 4.82 (yellow arrow); d-f: left adrenal gland, SUVmax = 5.14 (red arrow); CON = 1.07. 40 min ^68^Ga-pentixafor PET lateralization result (A-F) of patient 6 showed UPA (right), and AVS indicated BPA. A-C: right adrenal gland, SUVmax = 20.79 (yellow arrow); D-F: left adrenal gland, SUVmax = 7.87 (red arrow); and CON = 2.64.


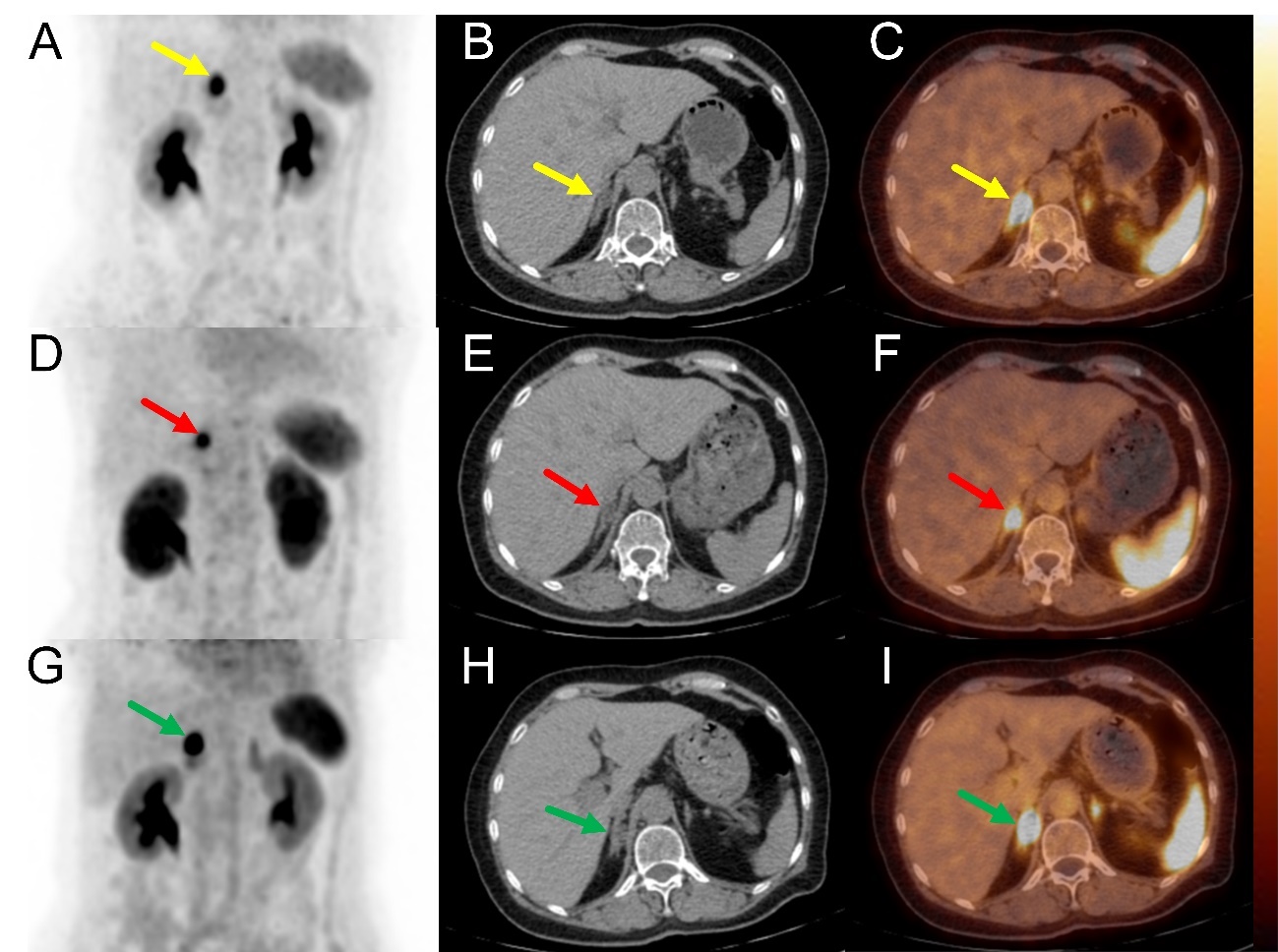
 Supplementary figure 3. The 10-min ^68^Ga-pentixafor PET/CT (A-C) and AVS of patient 17 showed UPA (right), SUVmax = 17.90 (yellow arrow), CON = 3.31. And potassium 3.5 mmol/L and ARR 62.12 [ng/dL]/[ng/mL/h]. After SAAE on the right, the patients were given PET (D-F) again on the second day, and 10-min SUVmax = 9.97 (red allow), CON = 2.80. And potassium 3.5 mmol/L and ARR 35.19 [ng/dL]/[ng/mL/h]. Three months later, the patients were followed up for potassium 3.3 mmol/L, ARR 206.70 [ng/dL]/[ng/mL/h] and blood pressure 139/91 mmHg. PET was performed again, the 10-min SUVmax = 26.02 (green arrow), CON = 6.00, followed by medication, resulting in clinical and biochemical persistence.


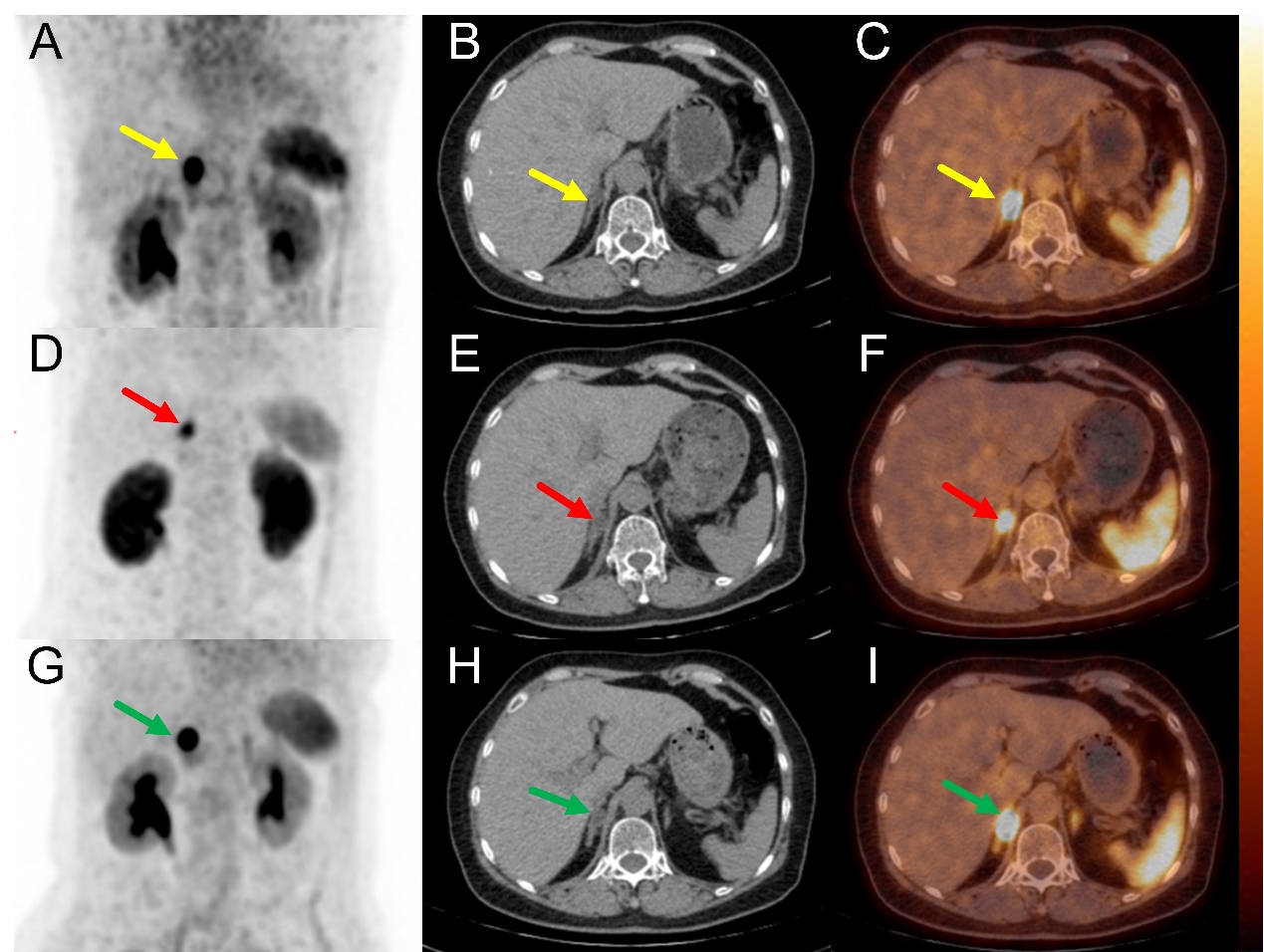


Supplementary figure 4 The 40-min ^68^Ga-pentixafor PET/CT (A-C) and AVS of patient 17 showed UPA (right), SUVmax = 18.11 (yellow arrow), CON = 5.17. After SAAE on the right, the patients were given PET (D-F) again on the second day, and 40-min SUVmax = 10.22 (red allow), CON = 3.25. PET was performed again after Three months, the 40-min SUVmax = 27.58 (green arrow), CON =4.70.
